# Osteochondral autograft transfer for post-traumatic osteochondral defects of the anterolateral surface of the distal tibial plafond

**DOI:** 10.1016/j.tcr.2016.05.008

**Published:** 2016-06-19

**Authors:** Hiromitsu Yabumoto, Yasuaki Nakagawa, Shigeru Yamada, Syogo Mukai, Seiji Mukaida, Shuzo Ninomiya, Naoya Tsubouchi, Masayuki Matsuoka, Eri Tarumi, Takashi Nakamura

**Affiliations:** Department of Orthopaedic Surgery, National Hospital Organization Kyoto Medical Center, 1-1 Mukaihata-cho, Fukakusa, Fushimi-ku, Kyoto 612-8555, Japan

**Keywords:** Ankle joint, Injury, Osteochondral defects, Tibial plafond, Osteochondral autograft transfer, Mosaicplasty

## Abstract

Post-traumatic osteochondral defects of the distal tibial plafond may be a more common cause of pain and osteoarthritis than previously recognized. However, the literature on the surgical treatment of osteochondral defects of the distal tibial plafond is significantly limited. This case report presents the operative technique and clinical outcome of osteochondral autograft transfer for an osteochondral defect on the anterolateral surface of the distal tibial plafond. A case of transfer of osteochondral autograft plugs to repair the anterolateral surface of the distal tibial plafond and prevent progression of forward displacement of the talus in a 25-year-old man who presented with pain in his right ankle, following a history of trauma.

## Introduction

The literature on the surgical treatment of osteochondral defects of the distal tibial plafond is very limited. These osteochondral defects may lead to pain and early degenerative changes. Recently, however, there have been several reports of the use of osteochondral autograft transfer (OAT) for the treatment of moderately sized defects in the knee [12], [14], [15], as well as for defects of the talar dome [10], [11]. Good operative results were reported in almost all cases. A case of post-traumatic osteochondral defect on the anterolateral surface of the distal tibial plafond and progressive osteoarthritis caused by forward displacement of the talus is presented. The patient was treated by transfer of osteochondral autografts to repair the anterolateral surface of the distal tibial plafond. This case report presents the operative technique and clinical outcome.

## Case report

The patient was a healthy 25-year-old man with a one-year history of right ankle pain following trauma.

He had met a car accident while walking. One year earlier, he had undergone open reduction and internal fixation on his right ankle for fracture at another hospital. A tibia diaphysis spiral fracture was fixed by the anterograde intramedullary nail with infra-patellar approach. An ankle malleolar fracture was fixed by the locking plate and cannulated cancellous screws with direct lateral and medial approach. He had finally consulted us because of worsening ankle pain while walking. On physical examination, there was tenderness in the anteromedial joint space of the right ankle. Slight ankle swelling was noted. Dorsiflexion of the right ankle was 10°, similar to that of the left ankle, but plantarflexion was restricted to 38°, compared with 60° on the left, but the ankle instability test was negative.

The first three months, even though we performed intra-articular injections, arthroscopic synovectomy for osteoarthritis, and the fixation implant removal in order to release implant irritation, his ankle pain persisted.

**Fig. 1:**
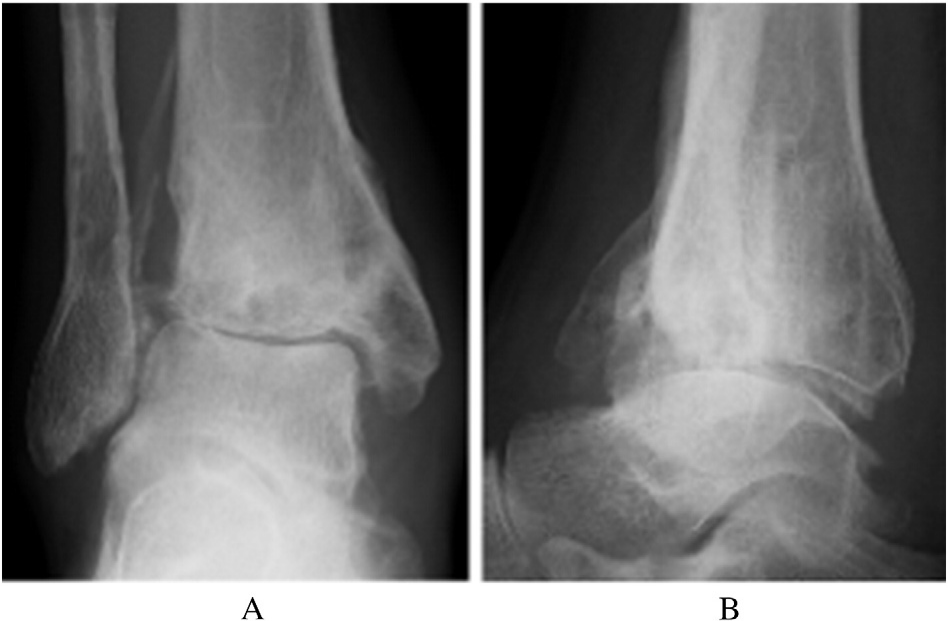
Radiographs at the initial examination showed an osteophyte in the anterolateral corner of the right plafond and moderate narrowing of the ankle joint. Forward displacement of the talus was seen: (A) anteroposterior view, (B) lateral view.

**Fig. 2:**
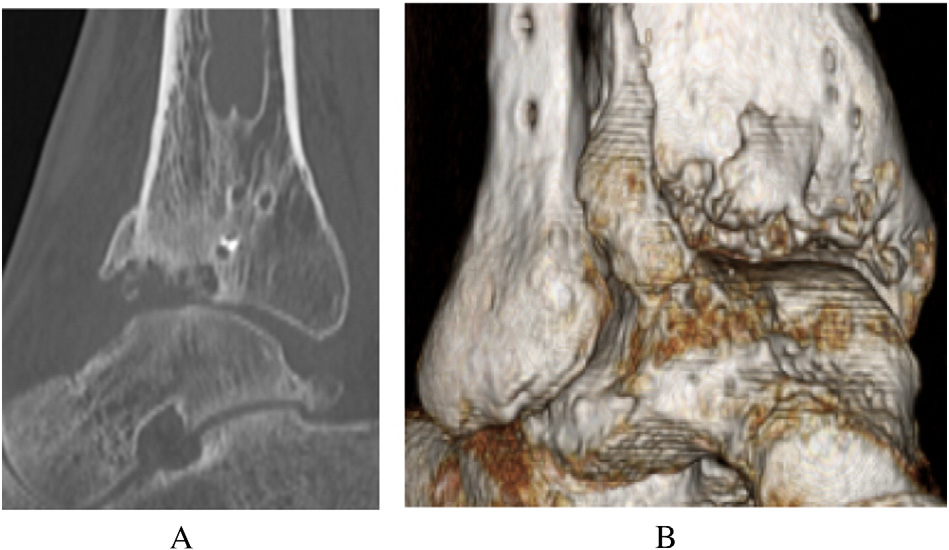
Computed tomography of the right ankle showed the osteochondral defect on the anterolateral surface of the distal tibial plafond, with a surrounding osteophyte: (A) 2D-CT sagittal view, (B) 3D-CT.

**Fig. 3:**
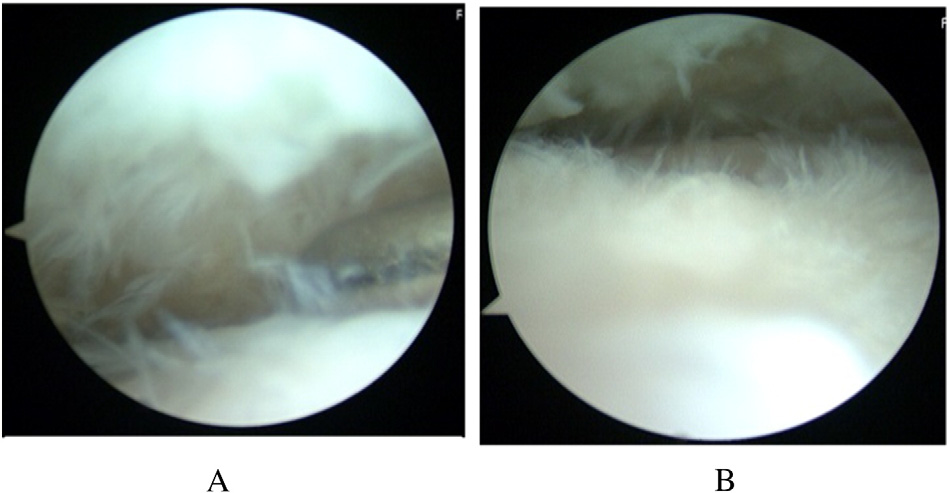
Arthroscopic examination showed a chondral lesion diagnosed as grade 2 according to the classification of the International Cartilage Repair Society over the entire ankle joint. The upper side was tibia, the lower side was talus. Arthroscopy was inserted in the anterolateral portal of his right ankle.

**Fig. 4:**
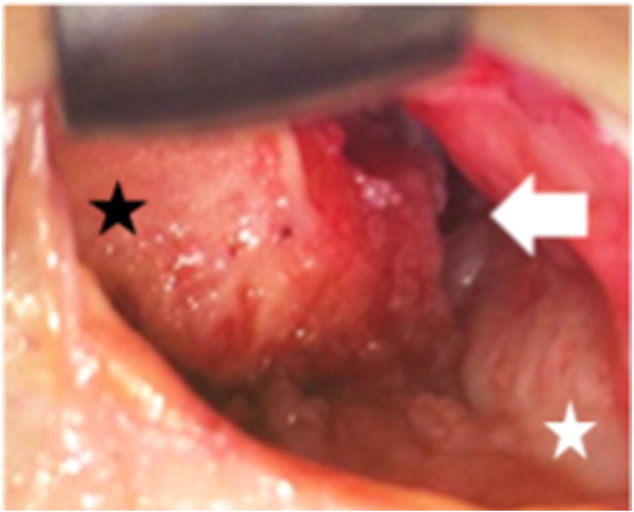
At surgery, the anterolateral plafond defect was measured 9 × 20 mm (arrow). The left side was proximal side, and the right side was distal side. Talus (white star). Tibia (black star).

**Fig. 5:**
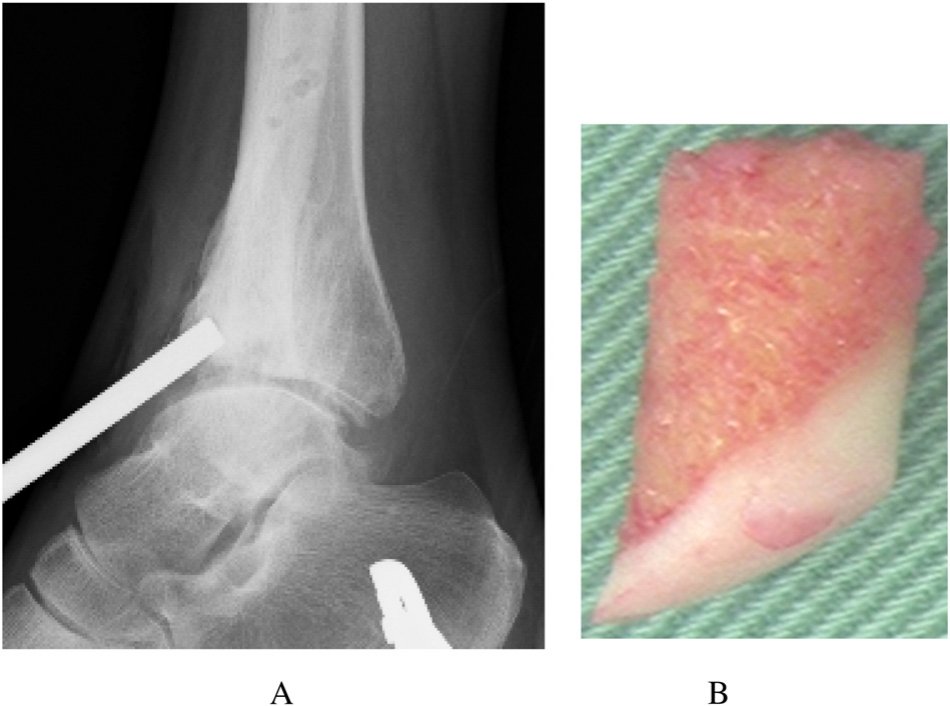
Based on a radiograph taken to calculate the inclination angle of the dilator, two 8-mm osteochondral autograft plugs ramped 35° and 45° were harvested: (A) radiograph at surgery, (B) a harvested plug ramped 35°.

**Fig. 6:**
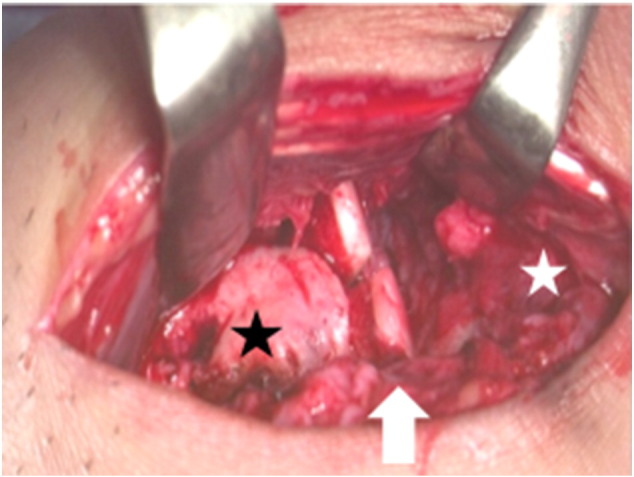
Surface of the anterolateral wall of plafond was almost completely smooth after OAT. Two grafted plugs into the recipient side (arrow). The left side was proximal side, and the right side was distal side. Talus (white star). Tibia (black star).

The AOFAS ankle score at that point in time was 50 [1]. Radiographs showed moderate narrowing of the ankle joint and forward displacement of the talus (Fig. 1-A, B). Computed tomography of the right ankle showed an osteochondral defect on the anterolateral surface of the distal tibial plafond (Fig. 2). This was diagnosed as progressive osteoarthritis caused by an osteochondral defect on the anterolateral surface of the distal tibial plafond, and surgical repair of the osteochondral defect was recommended. Three months later, the osteochondral graft was performed on the patient's right ankle. The patient was placed in the supine position under general anesthesia. The lower extremity was prepared and draped in the standard sterile fashion. We inserted the 2.0 mm K-wire in his right calcaneus, then skeletal traction was done in order to open his right ankle joint space if necessary. Next, 10 mL of fluid was injected intra-articularly to distend the ankle. The anterolateral ankle arthroscopy portal was established in the routine fashion, and global arthroscopy was performed. The osteochondral defect was identified about the anterolateral plafond which site was enthesis of the anterior tibiofibular ligament (Fig. 3), and osteochondral lesions of the tibia and talus were identified. An anteromedial portal was established. The osteochondral lesions were probed, and the osteochondral lesions were diagnosed as grade 2 according to the classification of the International Cartilage Repair Society [4]. We performed multiple OAT in the anterolateral plafond at the anterolateral wall with extension of the anterolateral portal. The anterolateral osteochondral defect was measured 9 mm × 20 mm (Fig. 4). The plug donor site was the lateral corner of the patellar groove in the right knee joint. Under direct vision, we harvested 2 bone plugs from the normal bone deeper than the osteochondral defect for the preparation of the recipient site in order to accept the osteochondral plugs from the donor site in maximum plantarflexion. Then, the dilator was interpositioned into the recipient site, and a radiograph was taken to calculate the inclination angle between the dilator and tibial plafond. Therefore, we thought that the smooth surface was obtained by the inclination of the grafted plugs. According to the angle required, two 8-mm osteochondral autograft plugs ramped 35° and 45° were harvested from the donor site (Fig. 5), and transferred into the anterolateral plafond at an angle to re-contour the articular surface and make it smooth. Both harvest and transplantation of osteochondral autograft plugs were performed by open approaches for transplantation in the exact slope. After this procedure, the surface of the anterolateral wall of plafond was almost completely smooth (Fig. 6). The depth of these plugs was 20 mm, deeper than the osteochondral defect. It was confirmed that the deepest area of the plugs consisted of a macroscopically normal bone. We used press-fit technique in order to stabilize the osteochondral plugs. Stabilization of the osteochondral fragments relied on tight interference with the host bone. In case of difficulty with press-fit technique, a screw made of polylactic acid and hydroxyapatite was prepared, but it was never used.

**Fig. 7:**
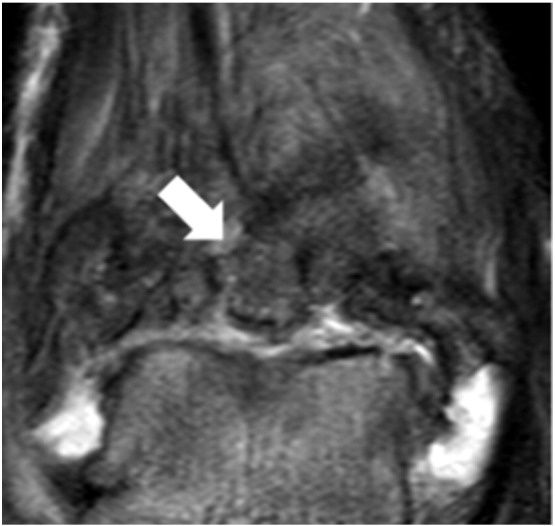
T2-weighted MRI showed an area of heterogeneous intensity (arrow).

**Fig. 8:**
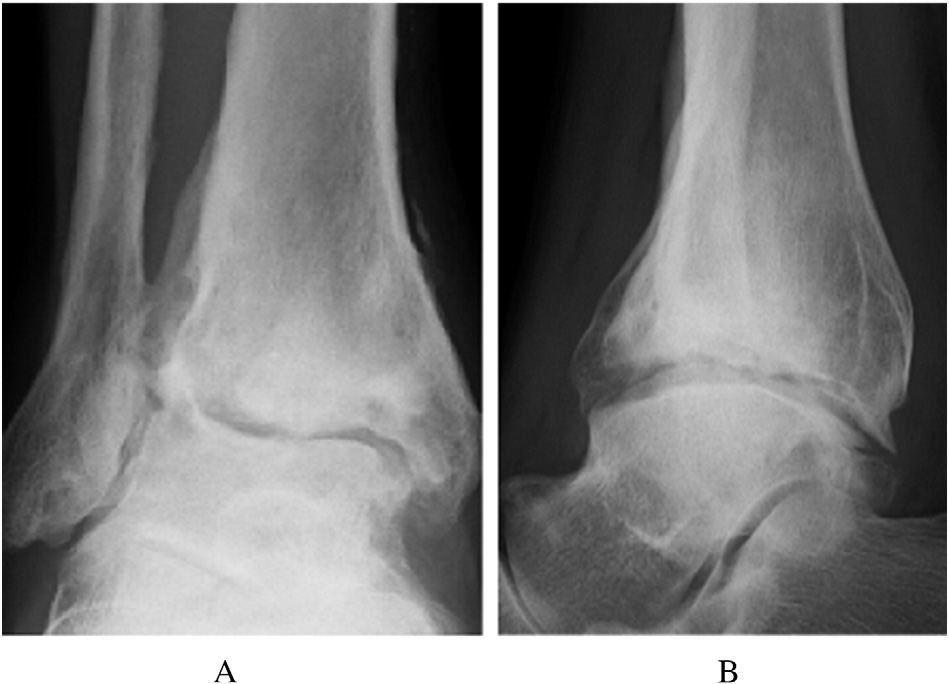
Radiographs obtained at 3 years after surgery showed an osteophyte in the anterolateral corner of the right plafond that was larger than that evident in the radiographs taken before OAT; forward displacement of the talus was not seen, and the joint space was preserved: (A) anteroposterior view, (B) lateral view.

Postoperatively, the foot was placed in a short leg cast, and the patient was advised to ambulate non-weightbearing for a 4-week period. The patient had an uneventful postoperative course, and a range of motion exercises were initiated immediately after cast removal at 4 weeks, with partial progressive weightbearing allowed during the next 4 weeks. The patient was able to return to standing work 10 months after surgery. The most recent magnetic resonance imaging scans at 2 years after surgery showed that the cartilage in the anterolateral joint surface was almost intact, and there was no osteonecrosis (Fig. 7). At 3 years the post-operative X-rays of the ankle joint depicted a larger osteophyte, compared with the pre-operative situation, in the anterolateral corner of the right tibial plafond. No forward displacement of the talus was evident on the lateral view of the right ankle joint, while the respective joint space was preserved (Fig. 8). On the latest physical examination, the patient complained of mild pain in his right ankle while walking, but could walk for 30 min. Dorsiflexion of the right ankle was 0°, compared with 10° on the left, and plantarflexion was 30°, compared with 60° on the left. The postoperative AOFAS ankle score was 80. The patient remained asymptomatic with respect to the donor sites of the right knee. He was able to return to carpenter's work on a five-day week, but remained with minor ankle pain on standing up and walking for more than half an hour over the 3-year observation period.

## Discussion

Most osteochondral defects of the tibial plafond are post-traumatic [5], [6], [7], [9], [11], [13], [18]. Such a post-traumatic lesion is an antecedent factor for the development of degenerative changes in several reports [3], [13], [16], [17]. OAT in the knee joint is well established and has good results for the treatment of osteochondral defects, and the results of treatment of talar dome lesions have suggested that OAT has good long-term clinical outcomes [8]. However, only a few cases of using OAT for osteochondral defects of the tibial plafond have been reported in the literature [18]. This case report provides an important clinical insight. OAT for post-traumatic osteochondral defects on the anterolateral surface of the distal tibial plafond can prevent secondary joint destruction. Hangody et al. [11] presented 2 to 7-year results of 36 patients after OAT for the treatment of osteochondral defects of the talus. Measured by the Hannover scoring system, they achieved good to excellent results in 34 cases (94%). Baltzer et al. [2] used OAT for osteochondral defects of the talus. They reported an average improvement according to the Evanski and Waugh score from 52 of 100 points preoperatively to 88 of 100 postoperatively, over a follow-up period of 2 years. Al-Shaikh et al. [1] reported favorable results using OAT for 19 patients with symptomatic osteochondral defects of the talus with mean follow-up of 16 months. The average postoperative AOFAS ankle score was 88, and most patients had occasional mild pain, but excellent function, range of motion, stability, and alignment. They used OAT to resurface osteochondral defects and achieved acceptable clinical results for most osteochondral defects. However, in osteochondral defects on the anterolateral wall of the plafond, it is necessary to not only resurface, but also ensure that the surface of the distal tibial plafond prevents progression of forward displacement of the talus and secondary joint destruction.

To form a joint surface smoothly is very important in order to achieve satisfactory results. Therefore, we harvest plugs obliquely as needed. In order to harvest the inclined plugs, the intraoperative radiography is a useful method. To the best of our knowledge, no paper has described OAT for osteochondral defects on the anterolateral surface of the distal tibial plafond. For the treatment of post-traumatic osteochondral defects on the anterolateral surface of the distal tibial plafond, OAT is the only treatment to fill the defect with intact bone and hyaline cartilage and prevent secondary joint destruction.

We think our procedure prevented severe progression of the osteoarthritis in our patient's right ankle. However, there were some opinions in the following; there was the progression of the osteoarthritis, following our procedure, with further limitation of the ankle range of motion, more osteophytes compared to previous images, but less pain. These results do not differ significantly from those following a formal ankle fusion.

We think that the best candidate may be the focal osteochondral defect in young and middle-aged patients. We think that arthroplasty is unfavorable for young and middle-aged patients by reason of its poor long-term outcome. Moreover, our procedure is better at residual range of motion than arthrodesis. The residual function allows patients to feel easy to walk.

This technique may provide an effective option for preventing secondary joint destruction caused by post-traumatic osteochondral defects on the anterolateral surface of the distal tibial plafond; however, further analysis is needed as only short- to medium-term follow-up data have been presented.

## Conflict of interest

There are no conflicts of interest from any of the authors.
